# Development of Tin Oxide-Based Nanosensors for Electronic Nose Environmental Applications

**DOI:** 10.3390/bios9010021

**Published:** 2019-02-05

**Authors:** Isabel Sayago, Manuel Aleixandre, José Pedro Santos

**Affiliations:** Institute of Physics Technology and Information (ITEFI-CSIC), 28006 Madrid, Spain; i.sayago@csic.es (I.S.); manuel.aleixandre@csic.es (M.A.)

**Keywords:** nanofibres, tin oxide, electronic nose, NO_2_, pollution, electrospinning, low detection temperature

## Abstract

Tin oxide nanofibres (NFs) are used as nanosensors in electronic noses. Their performance is compared to that of oxide commercial chemical sensors for pollutant detection. NFs were grown by electrospinning and deposited onto silicon substrates with integrated micro-hotplates. NF morphology was characterized by scanning electron microscopy (SEM). The NFs presented high sensitivity to NO_2_ at low temperature.

## 1. Introduction

Pollution monitoring is the key to air quality management. The concentration of air pollutants is measured at network reference stations using precise analytical instruments consisting of bulky, heavy, and difficult-to-use high energy-consumption equipment. Thus, the number of available stations is limited due to high operation and maintenance costs. The stations are preferentially located in urban areas, although in many cases they are far from the main sources of pollution. However, in rural areas with few inhabitants and in remote or inaccessible areas, the measuring stations and therefore the pollution data are not available.

Currently, the most promising alternative for monitoring atmospheric pollutants is the use of electronic noses formed by a sensor array. The first step in the development of electronic noses for environmental applications is reducing the cost of sensors. These sensors are required, besides their low cost, to be autonomous, easy to use, reliable and accurate. Their size, weight, and energy consumption must also be reduced [1]. Resistive sensors of metal oxide semiconductors (MOX) are suitable candidates for the development of low-cost, high-performance sensors due to the simplicity of the physical magnitude involved in the measurement (resistance) and the high sensibility to toxic gases. In particular, nanostructured materials are the most appropriate strategy to minimize some of the current problems with gas sensors (lack of sensitivity, power consumption, and stability).

In this work, we present the development of tin oxide nanosensors for electronic noses (e-noses). The two main applications of e-noses in the environment are pollution and odour monitoring. Due to the increased interest in this field and in order to improve potential use of instrumental odour monitoring, including sensors or e-noses, a new working group (WG41) started in 2015 within the framework of the European Committee of Standardization (CEN/TC264 Air Quality). The objective of this group was to propose a new European standard for instrumental odour monitoring [2]. Applications of electronic noses in the environment can be found in several works, some based on MOX [3] or amperimetric commercial sensors [4]. Other types of e-noses are those based on surface acoustic wave (SAW) sensors [5]. Biomimetic artificial noses, including whole-cell olfactory receptor protein and odorant binding protein (OBP)-based biosensors are also being studied [6]. Portable devices are being developed for the measurement of urban pollution [7,8,9].

Gas sensors based on sensitive layers of one-dimensional metal oxide (1D) nanostructures have shown superior performance to bulk sensors due to their large surface area–volume ratio and their dimensions being comparable to the extent of the surface charge region [10,11,12]. Tin oxide is still the most important material used for the detection of atmospheric polluting gases, and its most outstanding characteristics with respect to other semiconductors are its high sensitivity at low temperatures and low cost. One-dimensional SnO_2_ nanostructures (nanowires, nanobelts, nanoribbons, nanofibres, etc) can be synthesized using several methods like laser ablation, chemical vapour deposition, electro-deposition, thermal evaporation, rapid oxidation and electrospinning [13,14,15,16].

Electrospinning is a simple, versatile and economic technique that allows fibres to be obtained at micro and nanometric scales [17,18]. The electrospinning process began to be employed in conventional organic polymers of high molecular weight [19] and in the last decade has been used for the preparation of semiconductor oxide fibres from polymer solutions incorporating metallic precursors [20,21,22]. The process involves the application of an electrostatic field to a polymer solution with a certain viscosity and when the electric field strength is greater than the surface tension, the polymer solution is expelled to a collector in the form of a fibre.

Nitrogen dioxide (NO_2_) is one of the major air pollutants, especially in large cities. NO_2_ is an oxidizing gas whose main emission sources are combustion processes (heating, power generation and engines in vehicles and ships). Its effect on human health can be both short-term (causing significant inflammation of the respiratory tract) and long-term (affecting organs such as the liver and spleen, systems such as the circulatory system and the immune system, which in turn leads to lung infections and respiratory failure) [23]. In addition, nitrogen oxides alter the environment by contributing to the acidification and eutrophication (excess nitrogen nutrients) of terrestrial and aquatic ecosystems, leading to a loss of life in animals and plants and changes in species diversity [24].

The NO_2_ exposure limit values recommended by the World Health Organization (WHO) [25] are shown in Table 1. These low concentration ranges cannot be detected by commercial sensors at low temperature.

The European Commission [26] has urged member states to implement air quality management plans that ensure compliance with the standards set by the EU air quality directive [27] no later than 2020. Air pollution monitoring is a key air quality management task, for which the Air Quality Directive (AQD) opts for a strategy based on a network of a limited number of fixed stations, equipped with precision analytical instruments, which has some drawbacks.

Measuring equipment is bulky, heavy, difficult to use, and consumes a lot of energy. Equipment costs, operation, and maintenance are high. In many cases, the stations are located far away from areas of high traffic density where the greatest local increases in air pollution occur. A small number of these stations dispersed in a city allows data to be obtained with hourly resolution, but at a small number of points. In emergency situations, decisions are based on real-time measurements or, in the absence of such measurements, on predictive models of the distribution of pollutants in the atmosphere, the usefulness of which depends on the degree of validation of the models. Thus, although stations accurately measure air pollution, their spatial representativeness and temporal resolution are insufficient to capture the spatial–temporal variability of air pollution.

Although the AQD does not consider sensors as reference instruments, it does open the door to the use of sensors for indicative measurements, for which it sets less restrictive quality objectives. It is estimated that the use of low-cost, low-consumption sensors that meet AQD quality standards for indicative measurements would allow a 50% reduction in the minimum number of stations [28]. The new generation of sensors finds application (unregulated) in sectors such as personal and community monitoring of air quality, traffic management, estimation of exposure to air pollution, R&D, and environmental education, in which there are numerous business opportunities.

In this work, two prototypes of electronic noses for environmental applications based on low-cost sensors are described. The low-cost sensors tested were nanostructured tin oxide materials (nanofibres) obtained by an economical and versatile process (electrospinning) and commercial sensors. The sensor responses to low concentrations of NO_2_ [29] in controlled air atmospheres are also presented and discussed. We obtained good responses even at room temperature.

These nanofibre-based tin oxide resistive sensors can be incorporated into an electronic nose and could be used for air quality control.

## 2. Materials and Methods

### 2.1. Materials

Polyvinyl alcohol (PVA) and tin chloride (II) pentahydrate (SnCl_4_·5H_2_O) were used as precursor materials and distilled water was used as a solvent. PVA with an average molecular weight of 80,000 g/mol and SnCl_4_·5H_2_O were supplied by Sigma–Aldrich Química (Madrid, Spain). 

### 2.2. Preparation of Precursor Solution

First, an aqueous PVA solution (11% wt.) was prepared by dissolving PVA in distilled water and heating at 80 °C, under stirring for 2 h. Next, SnCl_4_·5H_2_O was added and the solution was cooled to room temperature, with stirring during cooling.

### 2.3. Synthesis of Tin Oxide Nanofibres

The SnO_2_ nanofibres (NFs) were prepared by an electrospinning process. The precursor solution (PVA + SnCl_4_·5H_2_O) was loaded into a syringe equipped with a metallic needle. A positive voltage of 19 kV was applied to the needle tip and the metal collector was grounded. The solution flow rate was 2 µL/min and the distance between the needle tip and the collector (silicon substrate) was 6 cm. Details of the electrospinning system are described in a previous work [30]. All sensors were prepared in the same conditions and NFs were grown onto micromachined silicon substrates with integrated heaters that allowed the calcination of the nanofibres in the test cell. The NFs were calcined at 500 °C for 4 h in air, obtaining nanofibres of SnO_2_.

### 2.4. Experimental Setup of the E-Nose System

#### Electronic Noses

**Figure 1:**
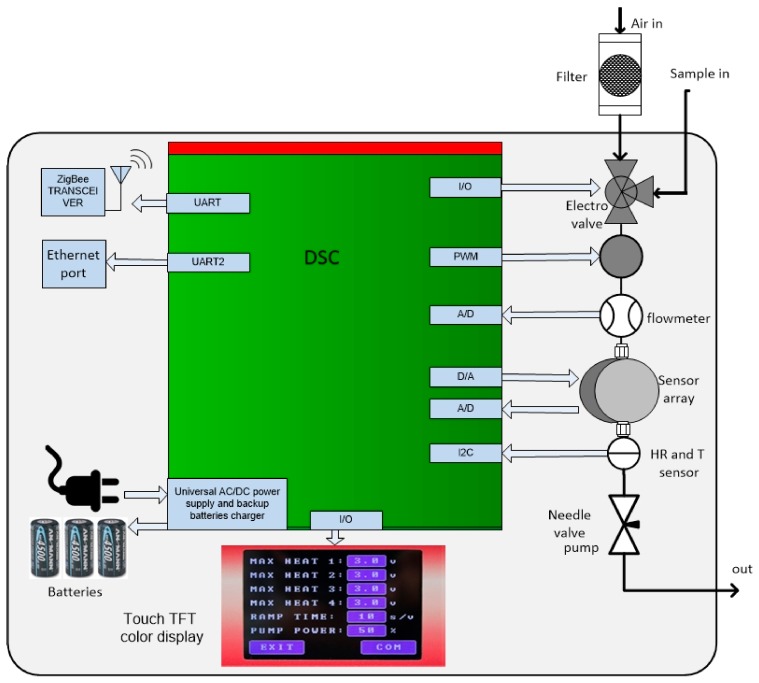
WiNOSE schematics.

Two electronic noses were developed: WiNOSE 5.0 for the nanosensors (R1, R2 and R3 nanofibre-based tin oxide sensors) and WiNOSE 6.0 for the commercial sensors. The schematics of both e-noses were very similar. The main difference between them is that the former is intended for laboratory use and the latter is a hand-held device that can also be used in the field [31]. Figure 1 shows the schematics of the WiNOSE. Details of the electronics can be found in [32].

**Figure 2:**
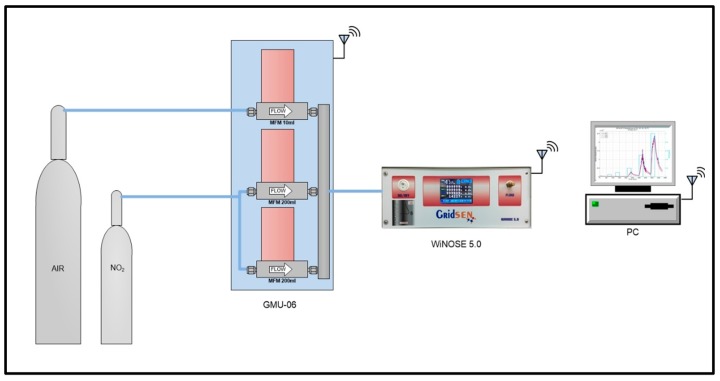
Scheme of the experimental design to measure the sensors.

The gases were generated by the dynamic dilution of bottles of 2 ppmv of NO_2_ in synthetic air. The sensors were heated to several temperatures using the resistances integrated into the micro-machined sensors and controlled by the electronic nose. The electronic nose and the gas generation instrumentation were controlled by a custom LabVIEW software that also registered the measurements to a computer. Figure 2 shows the scheme of the experimental set-up to measure the sensors.

Detections were carried out in air at temperatures ranging from 25 to 200 °C, with a constant flow of 200 mL/min. The NO_2_ concentrations varied from 0.1 to 0.5 ppmv with an exposure time of 10 min.

### 2.5. Sensor Tested

The WiNOSE 5.0 using three tin oxide NF nanosensors (R1, R2 and R3) was prepared with the same procedure. The silicon substrates of the sensors had integrated microheaters that allowed the sensitive layers to be heated and interdigitated electrodes (IDTs) to measure the sensitive layer resistance. The substrates with sensitive layers of tin oxide NFs covering the surface of the IDTs were mounted in a standard TO-8 package for the electrical characterization of the sensors. The TO-8 device was placed in the stainless-steel test cell inside the apparatus.

The WiNOSE 6.0 uses eight state-of-the-art commercial metal-oxide (MOX) microsensors, CC801 and CC803 (Cambridge CMOS Sensors Ltd., Cambridge, UK), operating at different temperatures. CC801 is intended for monitoring indoor air quality including carbon monoxide (CO) and a wide range of volatile organic compounds (VOCs), while CC803 is aimed at the detection of ethanol. However, like the majority of MOX sensors, they are also sensitive to NO_2_.

## 3. Results

### 3.1. Morphological Characterization of Tin Oxide Nanofibres

**Figure 3:**
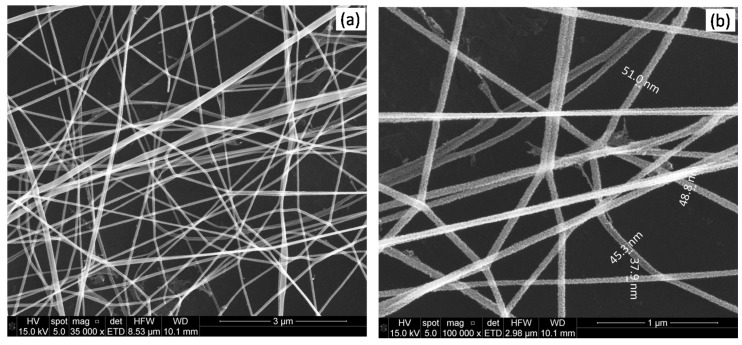
SEM micrographs of tin oxide NFs produced by electrospinning after calcination (500 °C in air for 4 h). (**a**) magnification 35000; (**b**) magnification 100000.

The fibres were randomly distributed on the substrate forming porous interlaced networks, as can be seen in the SEM images (Figure 3). In general, the fibres had nanometric diameters from 40 to 50 nm and their lengths reached several tens of microns. The nanofibres were constituted by multitude of nanograins whose diameters were less than 15 nm, as calculated from the broadening of the X-ray diffraction peaks in a previous work [30]. The nanograins were evenly distributed in the fibres, forming a porous nanostructure (Figure 3b). 

### 3.2. WiNOSE 5.0

**Figure 4:**
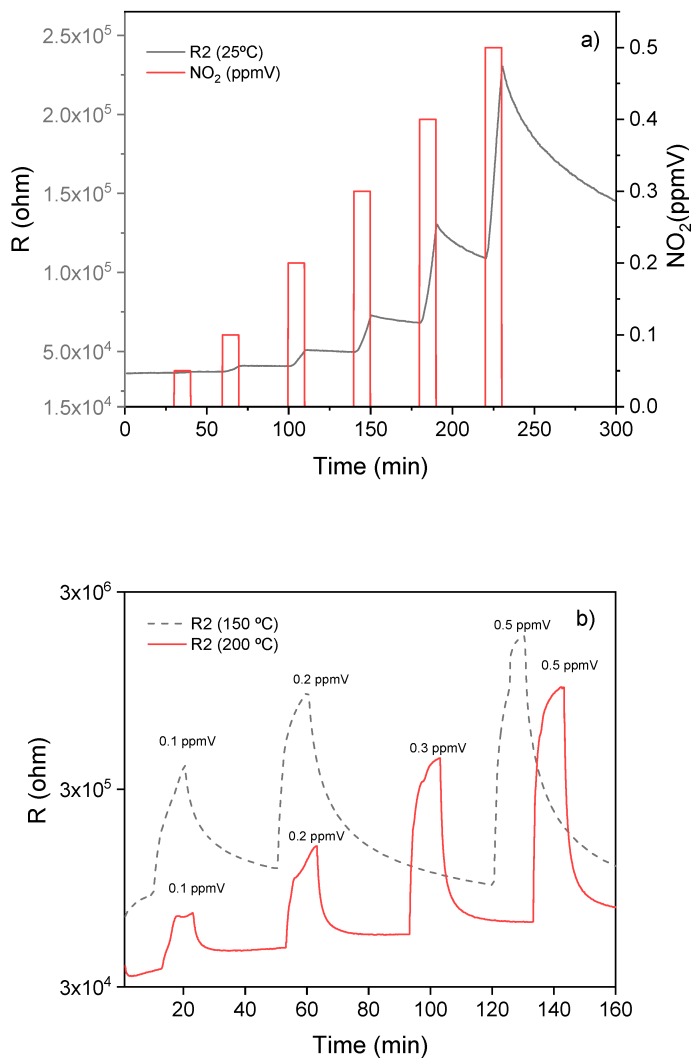
Response curves of the R2 sensor to NO_2_ at different temperatures: (**a**) room temperature and (**b**) 150 and 200 °C.

The WiNOSE 5.0 consists of the three tin oxide NF nanosensors (R1, R2 and R3). The sensors were exposed to different NO_2_ concentrations in the sub-ppmv range (0.1 to 1 ppmv). Figure 4 shows the sensor resistance changes in the detection processes. At room temperature, the resistance changed only with concentrations higher than 0.1 ppmv NO_2_. However, at 150 and 200 °C, the sensor detected 0.1 ppmv NO_2_ with a response (R = (R/R_a_), where R_a_ and R stand for the sensor resistance in air and under exposure to NO_2_, respectively) of 1.42 and 1.37, respectively. While the responses were high at temperatures below 200 °C, the response times were slow. At low temperature, the sensors did not reach saturation during the exposure time to NO_2_, although the resistance changes were observed after 2 min of exposure. Both the response and recovery processes depended on the operating temperature. At 200 °C, the responses obtained were lower than those reached at 150 °C. However, the sensors reached saturation at 200 °C during exposure to NO_2_ and at this temperature, the response and recovery times were lower than at 150 °C.

**Figure 5:**
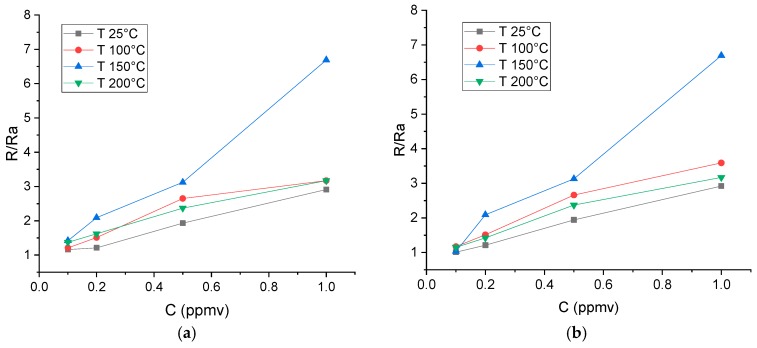
Response of the R1 (**a**) and R2 (**b**) sensors (sensitive layer of tin oxide NFs) to low NO_2_ concentrations at different operating temperatures.

Figure 5 shows the responses achieved in the detection of 0.1, 0.2, 0.5, and 1 ppmv NO_2_ at different temperatures with the R1 and R2 sensors. No remarkable differences were observed. The response of the R3 sensor was very similar to that of the other two. All sensors tested had a maximum sensitivity at 150 °C. Therefore, the optimum detection temperature may be between 150 and 200 °C.

**Figure 6:**
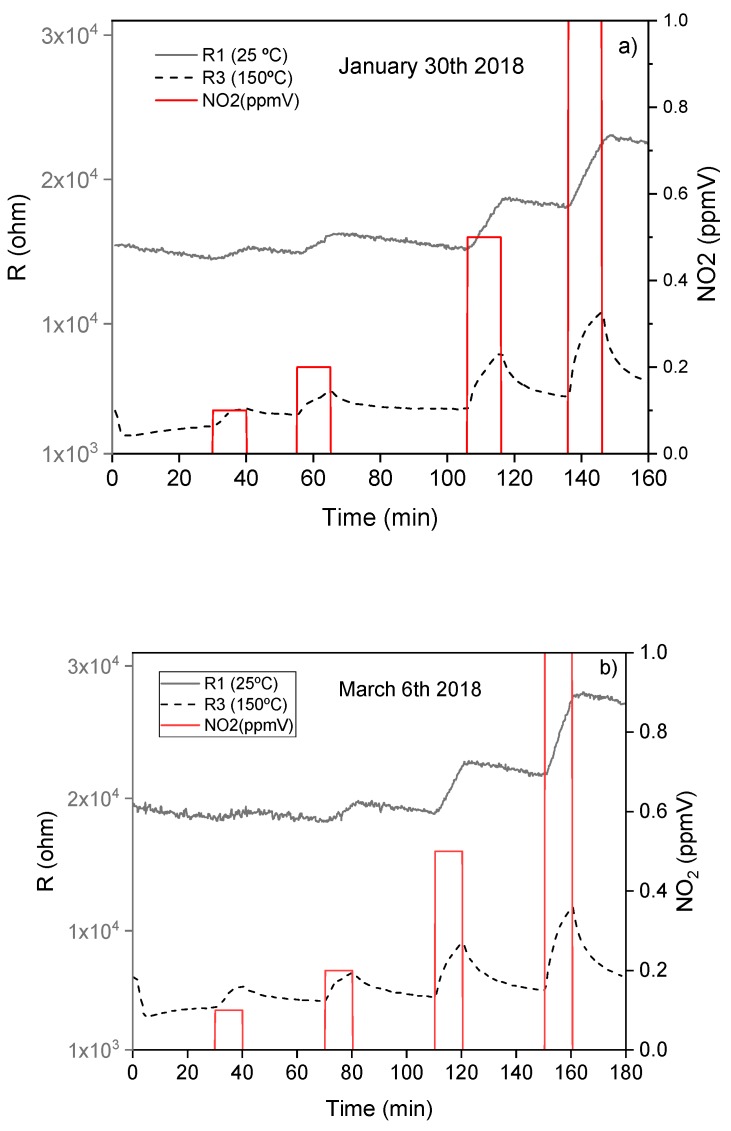
Repeatability of the R1 and R3 sensors exposed to different concentrations of NO_2_. Response curves of R1and R3: (**a**) initially, (**b**) after 10 weeks.

In order to check the long-term repeatability and reliability of the sensors, the detections were repeated after 10 weeks. The response curves obtained after inactive periods were similar (Figure 6), which confirms the reproducibility of the results. At 10 weeks, an increase of the sensors’ resistance was observed due to a slow aging process via interaction with surrounding gases. These increases were more evident as the operating temperature of the sensor became higher.

#### Calibrations of Nanosensors

**Figure 7:**
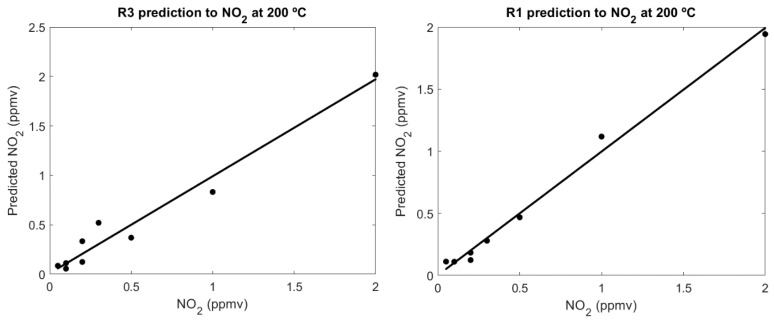
Calibration of the R1 and R3 sensor at 200 °C.

The responses of the R1, R2 and R3 sensors to NO_2_ were measured at 25 °C, 50 °C, 100 °C and 200 °C to determine the best operating temperature and the possibility of operating these sensors at low temperatures. In order to study more accurately the performance of the sensors, calibration curves were calculated and analysed for various temperatures. The responses of two of the sensors (R1 and R3) tested for NO_2_ detection (between 0.1 and 2 ppmv) were used for calibration. An ortho-normal calibration [33] was performed and the RMS and R^2^ of the calibrations were calculated, as shown in Figure 7. This calculation was repeated for each sensor and each temperature, and the results are compiled in Table 2. To test the combined power of the two sensors, we also carried out a partial least squares (PLS) regression with both sensors as independent variables and the concentration of NO_2_ as the dependent variable. The PLS was validated and evaluated by leave-one-out cross validation. This validation consisted of a loop in which every point was selected once. Then the rest of the points were used to compute a calibration that was used to predict the concentration of the point left out. This prediction was compared with the real concentration. The results can also be seen in Table 2.

### 3.3. WiNOSE 6.0

**Figure 8:**
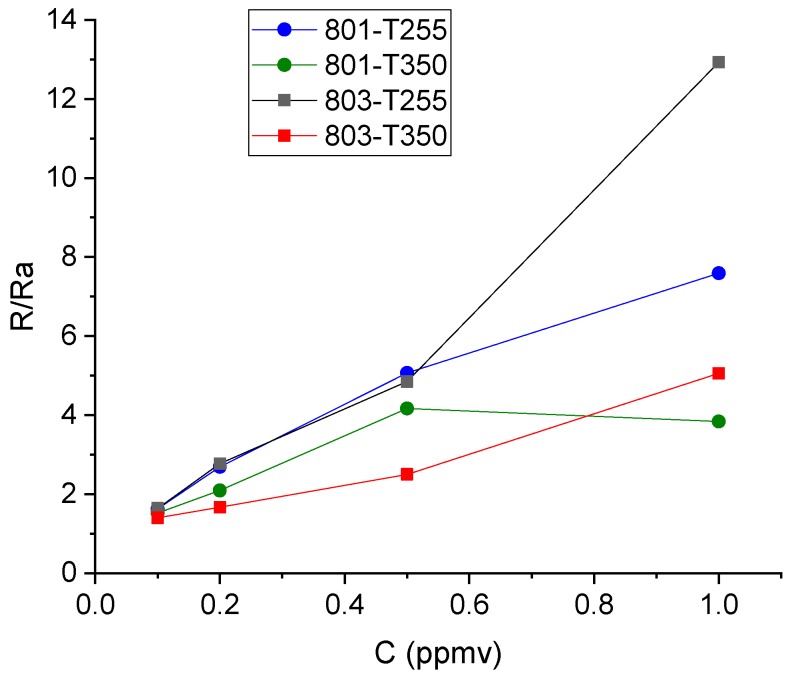
Response of the commercial sensors to low NO_2_ concentrations at two operating temperatures.

Measurements of low NO_2_ concentrations were performed at several temperatures ranging from 20 °C to 350 °C. Meaningful responses were only obtained above 250 °C. Figure 8 shows the response of the two types of commercial sensors at 255 °C and 350 °C. The same analysis as for the calibration was carried out for the commercial sensors and the results are summarized in Table 3.

## 4. Discussion

In the detection processes, the resistance changes occurred with the adsorption of gaseous molecules on the sensitive surface. Nanostructures were considered for gas detection applications due to their high surface area–volume ratio. In this work, the nanostructures—porous nanofibre networks—were composed of many nanograins that favoured the adsorption of gases.

The sensor calibrations had low errors, especially around 50–100 °C, and a lower error at room temperature. At higher temperatures, the sensors probably experienced some instability and the measurements had a much higher variability, which reflected the weaker performance. The sensors showed a good linear response in the concentration range tested. The combination of both sensors in a multilinear calibration was validated and the results were better estimated because the stricter validation and the aggregation of both sensors on a single performance was validated. The PLS had low error that tended to increase with the temperature and showed very good performance at 50 °C.

The sensors based on nanofibres had better low-temperature performance than commercial sensors and also better than that reported in the literature (Table 4). The references showed that NO_2_ concentrations lower than 0.5 ppm were detected and that the sensors would operate at moderate temperatures generally higher than 150 °C. Most of the references of the sensitive layers corresponded to complex nanostructures prepared by hydrothermal methods (due to difficulty to control the process, and problems of reliability and reproducibility). Although there are usually references for the sensor response (R_NO2_/R_air_), there is no detail of the sensor resistance. The commercial sensors used in this work, did not have any significant response below 250 °C, but they showed a more stable response with lower errors in the calibration for higher temperatures.

## 5. Conclusions

The results confirm that electrospun tin oxide nanostructured sensors can be used as sensors in electronic noses for environmental applications due to their high response to low NO_2_ concentrations, even at room temperature. They will allow for the development of new low-cost, low-consumption, sensor-based smart systems for the detection of gases. The adequate distribution of sensor networks (electronic noses) can provide information on pollution variation in large areas.

In future work, the humidity effect and ozone interference on sensor responses will be studied. In order to improve the sensor performance, catalytic metals (Au, Pd, and Ag) or graphene will be incorporated into the nanofibres. These additives will increase the sensor response at low temperatures and accelerate the processes of absorption and desorption.

## Figures and Tables

**Table 1:** NO_2_ limit values recommended by the WHO.

Average Annual	Average Hourly
40 µg/m^3^ (0.02 ppm)	200 µg/m^3^ (0.11 ppm) not to exceed more than 18 h per year

**Table 2:** Errors of the different calibrations.

T (°C)	RMS R1	RMS R3	RMS PLS	R^2^ R1	R^2^ R3	R^2^ PLS
25	0.186	0.0288	0.328	0.993	0.997	0.992
50	0.159	0.090	0.140	0.989	0.989	0.998
100	0.106	0.059	0.267	0.998	0.987	0.979
150	0.539	0.025	0.473	0.999	0.995	0.969
200	0.119	0.246	0.772	0.954	0.996	0.840
250	0.321	0.034	0.176	0.903	0.999	0.975
300	0.345	0.227	2.212	0.976	0.964	0.864

**Table 3:** Errors of the different calibrations for the commercial sensors.

T (°C)	RMS S801	RMS S803	RMS PLS	R^2^ S801	R^2^ S803	R^2^ PLS
255	0.008	0.043	1.197	0.990	0.987	0.941
350	0.372	0.006	0.695	0.949	0.989	0.984

**Table 4:** Comparison of NO_2_-resistive gas sensors based in nanostructured MOX.

Sensitive Layer	Concentration (ppm)	T (°C)	Response (R_NO2_/R_air_)	Ref.
In_2_O_3_ (nanorod clusters)	0.5	150	41	[34]
ZnO (nanowires)	0.5	225	18	[35]
SnO_2_ (nanowires)	0.5	200	17	[36]
SnO_2_ (hierarchical leaf-like)	0.5	65	7	[37]
SnO_2_ (nanofibrefibres)	0.1/0.5	25	1.16/1.93	This work
